# Exploring the Binding Mechanism and Dynamics of EndoMS/NucS to Mismatched dsDNA

**DOI:** 10.3390/ijms20205142

**Published:** 2019-10-17

**Authors:** Yanjun Zhang, Shengyou Huang

**Affiliations:** School of Physics, Huazhong University of Science and Technology, Wuhan 430074, Hubei, China; yjz@hust.edu.cn

**Keywords:** protein–DNA interactions, mismatch repair, EndoMS/NucS, molecular dynamics

## Abstract

The well-known mismatch repair (MMR) machinery, MutS/MutL, is absent in numerous *Archaea* and some *Bacteria*. Recent studies have shown that EndoMS/NucS has the ability to cleave double-stranded DNA (dsDNA) containing a mismatched base pair, which suggests a novel mismatch repair process. However, the recognition mechanism and the binding process of EndoMS/NucS in the MMR pathway remain unclear. In this study, we investigate the binding dynamics of EndoMS/NucS to mismatched dsDNA and its energy as a function of the angle between the two C-terminal domains of EndoMS/NucS, through molecular docking and extensive molecular dynamics (MD) simulations. It is found that there exists a half-open transition state corresponding to an energy barrier (at an activation angle of approximately 80${}^{\circ }$) between the open state and the closed state, according to the energy curve. When the angle is larger than the activation angle, the C-terminal domains can move freely and tend to change to the open state (local energy minimum). Otherwise, the C-terminal domains will interact with the mismatched dsDNA directly and converge to the closed state at the global energy minimum. As such, this two-state system enables the exposed N-terminal domains of EndoMS/NucS to recognize mismatched dsDNA during the open state and then stabilize the binding of the C-terminal domains of EndoMS/NucS to the mismatched dsDNA during the closed state. We also investigate how the EndoMS/NucS recognizes and binds to mismatched dsDNA, as well as the effects of K${}^{+}$ ions. The results provide insights into the recognition and binding mechanisms of EndoMS/NucS to mismatched dsDNA in the MMR pathway.

## 1. Introduction

For all living organisms, the fidelity of DNA replication is crucial for accurate transfer of generic information between generations. Unfortunately, some endogenous and environmental factors may cause uncorrected errors, which may lead to mutations potentially causing cell death, cancer, and neurodegenerative diseases [1]. Therefore, during evolution, organisms have developed several repair systems to preserve the genome integrity, such as nucleotide excision repair (NER) and base excision repair (BER), DNA mismatch repair (MMR), homologous recombination repair (HR), and non-homologous end joining [2,3,4,5,6,7]. MMR is responsible for correcting base substitution mismatches and insertion-deletion mismatches generated during DNA replication in organisms [3,8,9]. Extensive studies have found that MutS, MutL, and their homologs are key players in the MMR process in *Bacteria* and *Eukarya* [10,11,12,13]. However, the well-known MMR machinery is absent in most archaeal species, including *Crenarchaeota*, a few groups of *Euryarchaeota*, and almost all members of bacterial phylum *Actinobacteria* [14,15,16,17,18]. Although no gene has been identified as encoding the MutS/MutL homolog and the genes encoding MutS/MutL homologs are dispensable in archaeal species, the rates and spectra of spontaneous mutations in these organisms are comparable to the prokaryotes which have the MutS/MutL mismatch repair system [19,20,21]. A recent study has identified a novel endonuclease, named EndoMS (endonuclease mismatch-specific), in the hyperthermophilic *Archaea*: *Pyrococcus furiosus* and *Thermococcus kodakarensis*, which recognizes mismatched bases in the DNA strand and cleaves both strands to produce 5${}^{\prime }$-protruding ends and suggests a novel MMR pathway in archaeal species that operates without the MutS/MutL homology [14,15,22,23,24,25].

EndoMS is the ortholog of NucS, which has been thought to be an ssDNA-specific enzyme [26]. However, the study showed that the cleavage activity of 50 nM of EndoMS/NucS was not detectable for 5 nM ssDNA. Compared with the cleavage activity of EndoMS/NucS to ssDNA, only 1.3 nM of the same enzyme could cleave 5 nM T/G mismatched dsDNA substrate [15,24]. MutS has been reported to bind to a broad range of mismatches and correct G/T, C/A, C/T, A/A, T/T, and G/G mismatches with similar efficiencies [11,27,28]. EndoMS/NucS has cleavage activity for G/T, G/C, T/T, T/C, and A/G mismatches, with a higher preference for G/T, G/G, and T/T mismatches, but almost no effect on C/C, A/C, and A/A mismatches, which suggests that EndoMS/NucS has a different binding mechanism for mismatched dsDNA [15,24]. When EndoMS/NucS was removed from the actinobacterium *Corynebacterium glutamicum*, a drastic increase of spontaneous transition mutations in the EndoMS/NucS deletion strain was detected [15]. The crystal structure revealed that EndoMS/NucS is a homodimer, which has a significant conformational change before and after binding to dsDNA [24]. The C-terminal domains of EndoMS/NucS move approximately 40 Å and rotate by about 100${}^{\circ }$ to bind to dsDNA; after which, the C-terminal domains tether the dsDNA [24]. During the binding, the conformations of N-terminal domains are stable. The canonical MMR or BER enzymes flip out mismatched bases and bend dsDNA for recognition. For example, these two kinds of enzymes bend dsDNA about 42${}^{\circ }$ and 62${}^{\circ }$ to recognize [24]. However, EndoMS/NucS shows a different mismatch recognition mechanism, in which the angle of the dsDNA in the EndoMS/NucS-DNA complex is close to a canonical B-form double helix. The mismatched dsDNA recognition mechanism and binding process of EndoMS/NucS should be further studied to help us understand the novel MMR pathway in many archaeal species.

In this paper, we performed all-atom molecular dynamics (MD) simulations in solution, along with molecular docking, molecular modeling, and energy calculations, to gain insights into the recognition and binding mechanism of EndoMS/NucS to dsDNA that has mismatched bases. It is shown that there is a transition state at an activation angle (approximately 80${}^{\circ }$) between the open state and the closed state of the two C-terminal domains of EndoMS/NucS. When the angle is larger than the activation angle, the C-terminal domains are free to move and prefer to stay in the open state. Otherwise, the C-terminal domains will interact with the mismatched dsDNA directly and converge to the closed state at the global energy minimum. It was also found that the hydrogen bonds and π–π interactions between the N-terminal domains and the mismatched dsDNA play a critical role in recognizing and anchoring the mismatched dsDNA. In addition, our simulations reveal a new K${}^{+}$ binding site in the middle of two mismatched bases, which is necessary for bridging the two bases.

## 2. Results and Discussion

### 2.1. Energy Curve of EndoMS/NucS from the Open State to the Closed State

**Figure 1:**
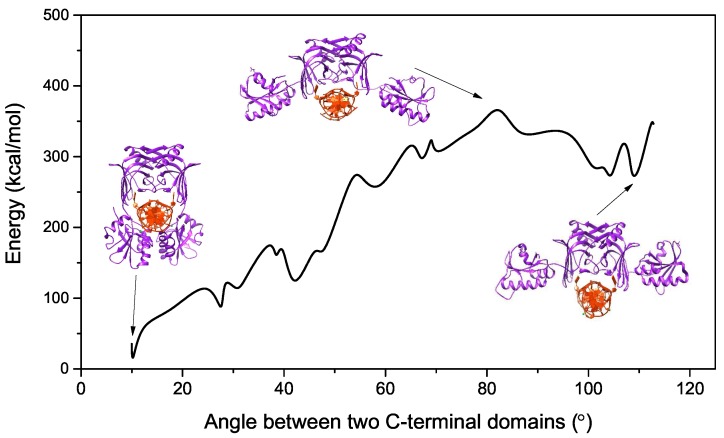
The relative energy of the EndoMS/NucS-dsDNA system as a function of the conformational co-ordinate (the angle between the two C-terminal domains of EndoMS/NucS).

The MutS is an important protein in the well-known MMR. It can bind to a broad range of mismatched bases and correct them with similar efficiencies [11,27,28]. The crystal structure of MutS indicates that it has flexible N-terminal domains to recognize mismatched dsDNA [29,30]. The movement of the highly flexible lever domains allows the DNA helix to enter the DNA-binding site. The new mismatched repair protein EndoMS/NucS shows a different binding process, which is consistent with restriction enzymes. According to the experimental structures of EndoMS/NucS in apo and dsDNA-bound forms, the C-terminal domains have to move about 40 Å and rotate by about 100${}^{\circ }$ to bind to the mismatched dsDNA [24]. In the binding process, the conformations of the N- and C-terminal domains do not show significant fluctuations. EndoMS/NucS shows repair activities preferably on G/T, G/G, and T/T mismatched bases, and has no effect on C/C, A/C, and A/A mismatched bases, which suggests a new recognition and binding behaviour [15,24]. It is necessary to explore the nature of the relevant energy to uncover the mechanism of the binding process of EndoMS/NucS to mismatched dsDNA. In order to eliminate the effect of conformational fluctuation of EndoMS/NucS, we constructed models of EndoMS/NucS-dsDNA from the open state to the closed state. The relative energy curve of the binding progress is shown in Figure 1. According to the energy curve of EndoMS/NucS-dsDNA, it can be seen that the closed state (at approximately 10${}^{\circ }$) is located at the global energy minimum, and the open state (at approximately 110${}^{\circ }$) is located at a local energy minimum, which indicates that the closed state is more stable than the open state. There is a energy barrier (at approximately 80${}^{\circ }$) between the open state and the closed state. Along the energy barrier to the closed state, there exist several energy minima. In the next section, we will give a detailed study of the binding process.

### 2.2. Binding Process of EndoMS/NucS to the Mismatched dsDNA

EndoMS/NucS is a new MMR protein identified in archaeal species, in which the well-known MMR machinery (MutS/MutL) is absent [14,15,22]. Due to the limitations of experimental techniques, the dynamic process of EndoMS/NucS binding to mismatched dsDNA is unclear. In this section, we detail the extensive MD simulations which were performed to explore the binding process of EndoMS/NucS to the mismatched dsDNA.

**Figure 2:**
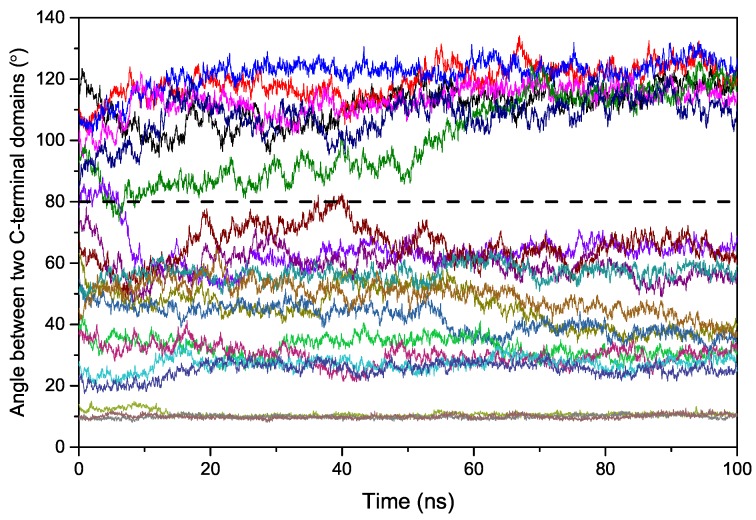
The angles between two C-terminal domains versus time for twenty systems (M1–M20).

**Figure 3:**
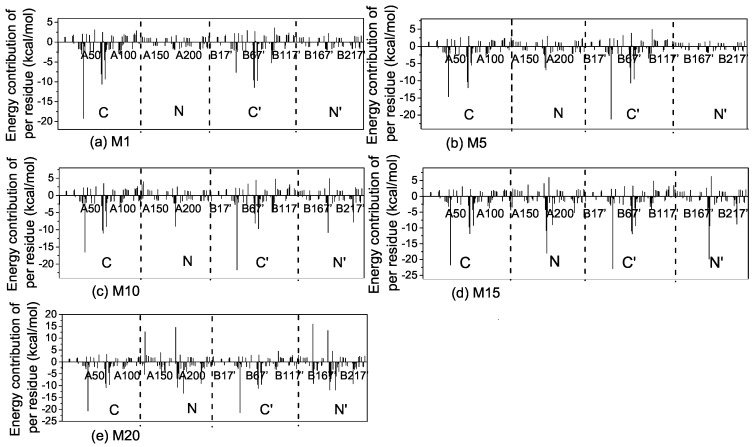
The interaction spectra of EndoMS/NucS with the mismatched dsDNA for (**a**) M1, (**b**) M5, (**c**) M10, (**d**) M15, and (**e**) M20. The interaction spectra of the other systems can be found in Figure S1.

We first performed short MD simulations (100 ns) on the 20 models at a temperature of 300 K to explore the binding process. In order to obtain insights into the conformational dynamics of EndoMS/NucS, we calculated the angles between two C-terminal domains versus time (Figure 2). As shown in Figure 2, the conformations of EndoMS/NucS around the angle 80${}^{\circ }$ have a big sparsity, which is consistent with the energy calculation. When the angle is larger than the activation angle (approximately 80${}^{\circ }$), the system shows a high mobility and tends to converge to the open state. This may be understood, because the open state is at the local energy minimum and there is no local energy minimum between the open state and the energy barrier (Figure 1). When EndoMS/NucS is in the open state, the C-terminal domains have direct interaction with the N-terminal domains, as revealed by analyzing the experimental structure [24]. This explains why the open state is stable when the C-terminal domains do not have interactions with the mismatched dsDNA. The energy barrier causes the open state to become relatively stable. When EndoMS/NucS is in the open state, the DNA-binding site on the N-terminal domains is exposed to solvent, which is important for EndoMS/NucS to recognize the mismatched dsDNA. When the angle is smaller than the activation angle, the C-terminal domains are relatively stable (Figure 2). Only M18 and M19 systems, for which the angle of the C-terminal domains is close to the closed state, converge to the closed state. Together with Figure 1, it can be found that other systems, such as M14 and M15, are trapped at a local energy minimum, and may need longer MD simulations to reach the closed state. We performed free energy decomposition on all the systems, based on a trajectory of 20 ns, and the results are shown in Figure 3 and Figure S1. Quantitative information about the binding free energy decomposition is very useful in identifying those residues that directly interact with the mismatched dsDNA. Figure 3 indicates that the interaction spectra between the N-terminal domains and the mismatched dsDNA are similar. The strong interactions between the N-terminal domains and the mismatched dsDNA indicate that the N-terminal domains can recognize the mismatched dsDNA and tightly catch it when EndoMS/NucS is in the open state. When the angle between the C-terminal domains is smaller than the activation angle (80${}^{\circ }$), the C-terminal domains of all these systems have direct interaction with the mismatched dsDNA. That explains why the angles between the C-terminal domains are stable when the initial angle is smaller than the activation angle. When the angle is smaller than the activation angle, the C-terminal domains are near the mismatched dsDNA, and easily interact with it. Therefore, the angle between the C-terminal domains is stable when the initial angle is smaller than the activation angle. We already know, from the free energy landscape, that the activation angle is located at an energy barrier, and the open state and the closed state are located at a local energy minimum and the global energy minimum, respectively. Based on the MD simulations, when the angle is larger than the activation angle, the C-terminal domains tend to move to the open state, which is in agreement with the energy landscape. However, the C-terminal domains may not quickly transit to the closed state when the angle is smaller than the activation angle, due to the existence of some local energy minima.

**Figure 4:**
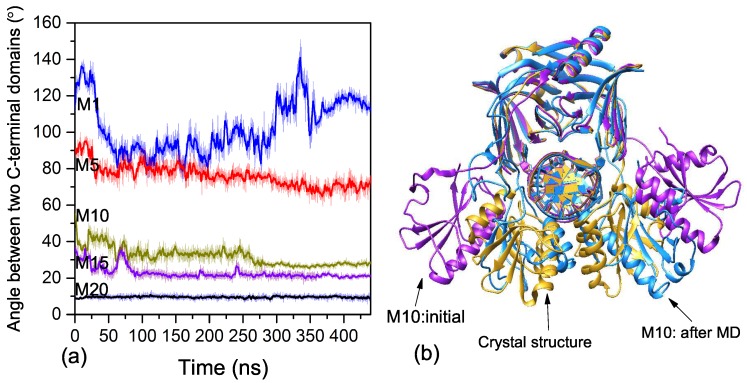
(**a**) The angles between two C-terminal domains of M1, M5, M10, M15, and M20 during long MD simulations. (**b**) The average structures during the last 2 ns, compared with the initial structure and active structure of M10. The initial structure of M10 is colored in purple; the crystal structure (the closed state) is colored in yellow; and the average structure after the MD simulation is colored in blue.

We also performed longer MD simulations on five selected models (M1, M5, M10, M15, and M20) under a higher temperature to further study the binding process of EndoMS/NucS to the mismatched dsDNA. For classical MD simulations, a lot of time is needed to explore the dynamic process of the C-terminal domains, so we performed these MD simulations at a higher temperature to increase the reaction rate. Figure 4a shows the angle curves of M1, M5, M10, M15, and M20 during the MD simulations. The average structure during the last 2 ns, compared with initial structure and active structure of M10, is shown in Figure 4b. As shown in Figure 4a, the angles of M1 show large fluctuations and the two C-terminal domains do not directly interact with the mismatched dsDNA.

The angles of M5, M10, and M15 show a significant decrease during the MD simulations. Only one C-terminal domain of M5 had an interaction with the mismatched dsDNA. For M10 and M15, in which the initial angles were smaller than activation angle, the two C-terminal domains first interacted with the mismatched dsDNA and then transitioned to the closed state. The average structures of M10 and M15 were close to the closed state, which further confirms speculation that the C-terminal domains directly interact with the mismatched dsDNA and then transition to the closed state (Figure 4b). The C-terminal domains need to overcome the energy barrier (activation angle) to interact with the mismatched dsDNA when the angle is larger than the activation angle.

### 2.3. Interactions of the Open State and the Closed State with Mismatched dsDNA

**Figure 5:**
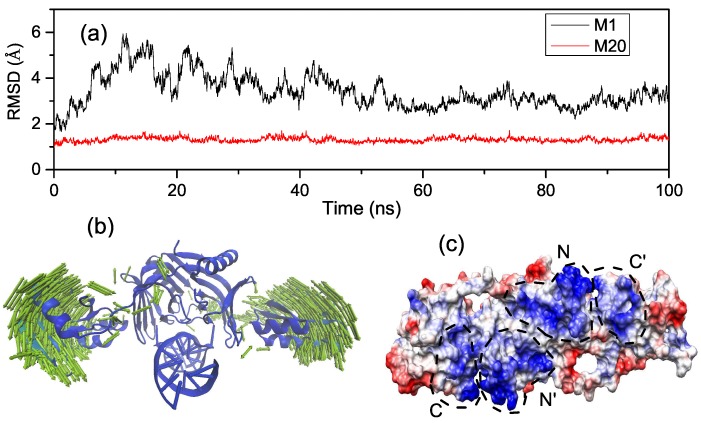
(**a**) The root mean square deviation (RMSD) of EndoMS/NucS C${}_{\alpha }$ atoms of the open state (M1) and the closed state (M20), relative to the starting structure, as a function of time. (**b**) Principal component analysis of M1. (**c**) Electrostatic surface representation of EndoMS/NucS (scale, −10 kcal/(mol·e) to +10 kcal/(mol·e), red to blue).

Exploring the interactions of the open and closed states with mismatched dsDNA can help us understand how EndoMS/NucS recognizes and anchors the mismatched dsDNA during the MMR process. The study of this section is based on short MD simulations of the open state (M1) and the closed state (M20). In order to explore the conformational stabilities of M1 and M20, the root mean square deviation (RMSD) of EndoMS/NucS C${}_{\alpha }$ atoms during the MD simulations, relative to the initial structure, were calculated; as shown in Figure 5a. Compared with that of M20, the RMSDs curve of M1 shows large fluctuations. From the PCA analysis, we find that the large fluctuation of M1 is mainly caused by the movement of two C-terminal domains (Figure 5b), as it is hard for the the C-terminal domains to interact with the mismatched dsDNA when the angle is larger than the activation angle.

To get detailed information about the interaction mechanism between EndoMS/NucS and mismatched dsDNA, the absolute binding free energies of M1 and M20 were calculated. The details about binding free energies of M1 and M20 are summarized in Table 1. The binding free energy of M20 (−122.94 kcal/mol) was about 2 times that of M1 (−68 kca/mol). The decomposition results of the binding free energy indicate that the contributions of the residues at the N-terminal domains of M1 and M20 were similar to each other, which indicates that the difference between the binding free energies of M1 and M20 is mainly because of the interaction of the C-terminal domains with the mismatched dsDNA (Figure 3a,e). The binding energy contributions of M1 come from the N-terminal domains, because only the N-terminal domains interact with the mismatched dsDNA when EndoMS/NucS is in the open state. The binding energy contribution (not including entropy contribution) of N-terminal domains of M1 and M20 are −114.62 and −146.22 kcal/mol, respectively. The large and stable binding energy between the N-terminal domains and the mismatched dsDNA of M1 and M20 suggests that the N-terminal domains play a critical role in recognizing and catching mismatched dsDNA. Table 1 also shows that the electrostatic interaction is important for the recognition and binding of EndoMS/NucS. We calculated the electrostatic surface potential of EndoMS/NucS and the result is shown in Figure 5c. The regions that directly interacted with the mismatched dsDNA show positive surface, which agrees with the calculation of binding free energy. The interface of the N-terminal domains shows more positive charges than the C-terminal domains. The positive surface is favorable to recognize and interact with the negative mismatched dsDNA.

**Figure 6:**
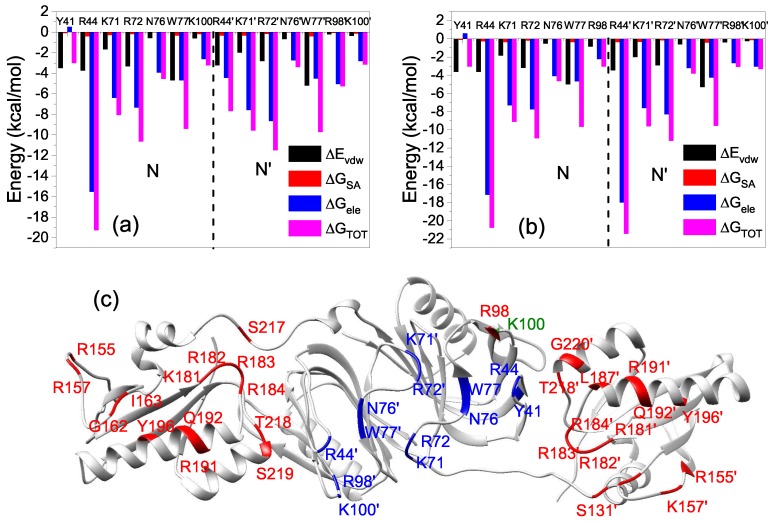
Residue-based decomposition of the binding free energy from the sum of electrostatic interactions and polar solvation energy (ΔG${}_{ele}$ = ΔE${}_{ele}$ + ΔG${}_{GB}$), the van der Waals energy (ΔE${}_{vdw}$), and nonpolar solvation energy (ΔG${}_{SA}$) for the key residues of N-terminal domains: (**a**) M1 and (**b**) M20. (**c**) The key residues that are important for EndoMS/NucS-dsDNA interactions. The residues belonging to M1 are colored in green, those belonging to M2 are colored in red, and those belonging to M1 and M20 are colored in blue.

**Figure 7:**
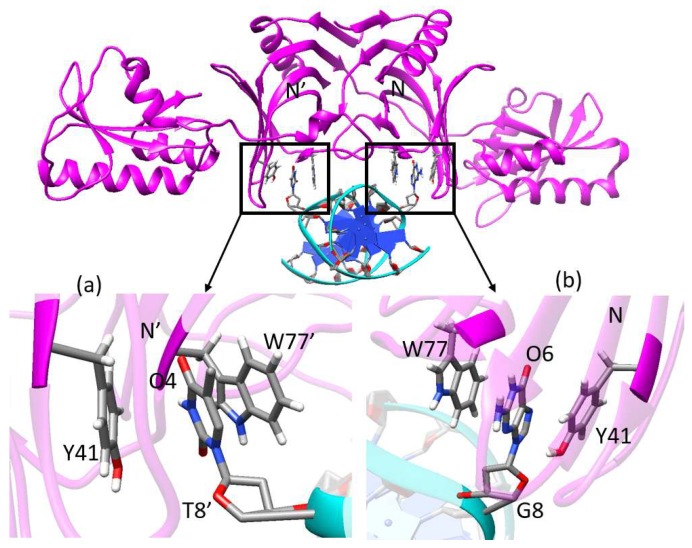
The recognition sites of EndoMS/NucS.

The residues that have a large contribution (≤−3 kcal/mol) to the binding free energy of N-terminal domains of M1 and M20 are represented in Figure 6, and detailed information about the energy contributions of key residues is listed in Table S1. The basic residues (Lys and Arg), which bring positive charges, are important for anchoring the mismatched dsDNA. The side-chains of Tyr41 and Trp77 (Tyr41${}^{\prime }$ and Trp77${}^{\prime }$), which form the base recognition sites located at the N-terminal domains, can form a π-stacking interaction with a G or T base (Figure 7). The mismatched bases, which are flipped out from the DNA double helix due to the weak interaction, insert in the middle of the two side-chains of Tyr41 and Trp77 (Tyr41${}^{\prime }$ and Trp77${}^{\prime }$). The residue-based decomposition of binding free energy for key residues located at N-terminal domains are shown in Figure 6. For the basic residues (Lys and Arg), the main driving force for the binding of EndoMS/NucS and the mismatched dsDNA is the pure electrostatic interaction (ΔG${}_{ele}$). The electrostatic interactions of basic residues come from the interactions of their side-chains containing amino groups with the mismatched dsDNA. The van der Waals (VDW) energies of the residues Tyr and Trp, which form the recognition sites, are the main contribution to the binding energy, due to the π–π interactions between their side chains and the mismatched bases. Compared with Figure 6a,b, it can be found that the energy contribution of Arg44${}^{\prime }$ is largely increased in the closed state. The increased energy contribution mainly comes from the electrostatic interaction of its side-chains and this is as its side-chain forms a new hydrogen bond with the mismatched dsDNA in the closed state (Section 2.4). The C-terminal domains of the closed state have strong interactions with the mismatched dsDNA. The contributions of the C-terminal domains also mainly come from the basic residues (Lys and Arg), as can be found in Figure S2 and Table S1.

### 2.4. Hydrogen Bonds Analysis

In order to investigate the influence of the configuration on the hydrogen bonding network, hydrogen bond length and occupancy for the M1 and M20 systems were calculated by the CPPTRAJ module of AMBER16 during the MD simulations, and the results are listed in Table 2 and Table S2. The M1 and M20 systems both form a complex hydrogen bond network with the mismatched dsDNA. The hydrogen bond networks between the N-terminal domains and the mismatched dsDNA of M1 and M20 were almost the same, except the for Arg44${}^{\prime }$ in M20 (Table 2). Arg44${}^{\prime }$ forms three new hydrogen bonds, OP2(T10)⋯NH1–HH12(Arg44${}^{\prime }$), OP1(T10)⋯NH2–HH22 (Arg44${}^{\prime }$), and OP1(G9)⋯NH1–HH11 (Arg44${}^{\prime }$), and the occupancies are 99.8%, 98.2%, and 78.6%, respectively.

The new hydrogen bonds of Arg44${}^{\prime }$ explain why the energy contribution of the side-chain of Arg44${}^{\prime }$ is greatly increased in M20. The residues that are located at the recognition sites can form stable hydrogen bonds with the mismatched bases, which are flipped out from the DNA double helix. The mismatched base T8 forms hydrogen bonds with Asn76${}^{\prime }$ and Trp77${}^{\prime }$, both in M1 and M20, with high occupancy (≥97%). The mismatched base G8${}^{\prime }$ also forms hydrogen bonds with the same residues Asn76 and Trp77 in the other recognition site, with high occupancy (≥99%). Trp77 not only forms a binding site with Try41 to recognize mismatched bases (G or T), but also forms the hydrogen bond with the mismatched base to anchor the mismatched dsDNA. The other hydrogen bonds are mainly formed between the basic residues and backbone of the mismatched dsDNA. The C-terminal domains of M20 also form a complex hydrogen bond network with the mismatched dsDNA, which causes the C-terminal domains to tightly catch the mismatched dsDNA (Table S2). Combined with the binding energy contribution of key residues, we find that the residues that form stable hydrogen bonds with the mismatched dsDNA also have a great binding energy contribution. It can be concluded that the hydrogen bonds play a critical role in recognizing and anchoring the mismatched dsDNA.

### 2.5. Ion Binding Sites

**Figure 8:**
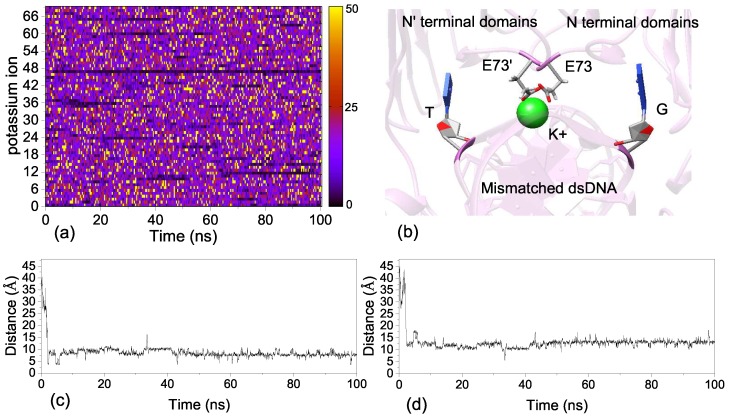
(**a**) The velocity changes of all K${}^{+}$ ions for M20 during MD simulations. (**b**) The K${}^{+}$ ion binding site in the dsDNA-bound system. (**c**) The distances between K${}^{+}$ and mismatched base G8 during MD simulations. (**d**) The distances between K${}^{+}$ and mismatched base T8’ during MD simulations.

The previous experiment found that the MMR function of EndoMS/NucS required metal ions (Mg${}^{2+}$) [24]. The crystal structure of dsDNA-bound includes two Mg${}^{2+}$ ions, and we kept these two Mg${}^{2+}$ ions in MD simulations. We explored the stability of the Mg${}^{2+}$ ions by calculating the distance of Mg${}^{2+}$ to the mismatched dsDNA; the results are show in Figure S3c,d. The Mg${}^{2+}$ binding sites are located in the middle of the C-terminal domains and the backbone of the mismatched dsDNA. The side-chains of two glutamate acids (such as Glu 132${}^{\prime }$ and Glu179${}^{\prime }$) and the phosphate group of the mismatched dsDNA form the Mg${}^{2+}$ ion binding sites (Figure S3a,b). It is known that the phosphate group of the mismatched dsDNA and the side-chain of glutamate acid both carry negative charges. The binding of Mg${}^{2+}$ can eliminate the unfavorable effects of glutamate acids on the binding of the mismatched dsDNA. The two Mg${}^{2+}$ ions are stable at the ion-binding sites during the MD simulations (Figure S3a,b). We also explored the movement of all K${}^{+}$ ions. Figure 8a shows the change of velocity of all K${}^{+}$ ions during the MD simulations. It can be found, from Figure 8a, that there is a stable K${}^{+}$ ion binding site in the dsDNA-bound system. The binding site of K${}^{+}$ ion is in the middle of two mismatched bases, which are both flipped out from the DNA double helix due to the weak interaction. The side-chains of Glu73 and Glu73${}^{\prime }$ are also involved in the formation of the K${}^{+}$ binding site (Figure 8b). We also calculated the distance between K${}^{+}$ and the mismatched bases G8 and T8${}^{\prime }$, and the results are shown in Figure 8c,d. It can be seen, from Figure 8c,d, that the K${}^{+}$ is free at the beginning of MD simulations and then is caught by the ion binding site at 2 ns. The K${}^{+}$ ion may play an important role in stabilizing the dsDNA-bound complex, because it can eliminate the disadvantageous effects of negative charges carried by glutamate acids on the binding of the mismatched dsDNA.

## 3. Materials and Methods

### 3.1. Protein Models Preparation

**Figure 9:**
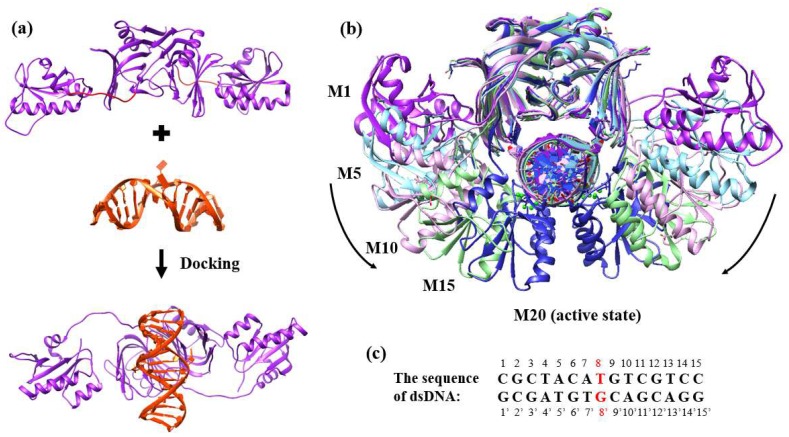
(**a**) The docking process of apo EndoMS/NucS with the mismatched dsDNA. The docking was based on the dsDNA-bound crystal structure (PDB: 5GKE). The missing loops of EndoMS/NucS, modeled by MODELLER, are colored in red. (**b**) Twenty models from the open state (M1) to the closed state (M20) were modeled by the Chimera software. Detailed information for all the models (M1–M20) can be found in Table S3. (**c**) The mismatched dsDNA sequence that was used in this study. The mismatched base pair (T/G) is colored in red.

The structure of the dsDNA-bound form of EndoMS/NucS was obtained from the Protein Data Bank (PDB: 5GKE), in which the dsDNA has a G–T mismatched base at position 8 [24]. The apo structure of EndoMS/NucS was also obtained from the PDB (PDB: 5GKJ) [24]. The missing loops of apo EndoMS/NucS were modeled by the MODELLER software (Figure 9a) [31]. All water molecules were removed from the PDB file. The Mg${}^{2+}$ ions were retained for all systems, as a previous experiment has revealed that the MMR function of EndoMS/NucS requires Mg${}^{2+}$ ions [24]. The open state of dsDNA-bound EndoMS/NucS was generated by docking the mismatched dsDNA onto the apo form of EndoMS/NucS [32,33,34,35,36], which was based on the dsDNA-bound crystal structure 5GKE (Figure 9a). We constructed the models of the dsDNA-bound state from the open state (apo form of EndoMS/NucS) to the closed state (dsDNA-bound form of EndoMS/NucS). The pathway of conformational translation was based on the angle between the two C-terminal domains, from the open state to the closed state (Figure 9b). Twenty models from the open state (M1) to the closed state (M20) were modeled by the Chimera software and the detailed information of all the models (M1–M20) can be found in Table S3 [37]. The residue numbers of EndoMS/NucS were referenced from the crystal structure of 5GKE; A: 5-237 and B: 5${}^{\prime }$-237${}^{\prime }$. The sequence of the mismatched dsDNA is shown in Figure 9c, where the mismatched bases are colored in red. The webserver H++ was used to determine the protonation states and add hydrogen atoms for all EndoMS/NucS-dsDNA models [38,39,40].

### 3.2. Molecular Dynamics Simulation Protocol

All MD simulations presented in this work were performed using the AMBER16 package [41]. The AMBER ff14SB force field was adopted for EndoMS/NucS-dsDNA structures and the Leap module was used to generate the topology and co-ordinate files. All the protein models were solvated in a cubic periodic water box of TIP3P [42] model with a cutoff of 12 Å. The solute was neutralized with potassium ions and then K${}^{+}$/ Cl${}^{-}$ ion pairs were added to reach a concentration of 150 mM. All systems were subjected to MD simulations with periodic boundary conditions. The cutoff value of non-bonded interaction was set to 10 Å. The long-range electrostatic interaction was calculated by the Particle Mesh Ewald (PME) method [43]. The SHAKE algorithm [44] was used to constrain all bonds involving hydrogen atoms.

All the MD simulations included two stages: Minimization and equilibration [45,46,47]. The minimization included three steps: The systems were first subjected to 2500 steps of steep descent movements, followed by 2500 steps of conjugate gradient minimization to remove the bad clashes between solute and solvent. Then, the systems were gradually heated from 0 K to 300 K in 50 ps. Finally, the systems were minimized at NVT ensemble for 50 ps. The atoms of protein and dsDNA structures were restrained by a harmonic restraint of 20 kcal·mol${}^{-1}$Å${}^{-1}$. Next, the systems were equilibrated using Langevin dynamics under constant temperature and constant-pressure (NPT) conditions at 300 K and 1 atm for 250 ps without any position restraints. Then, the production simulations were performed as an NPT (300 K, 1 atm) ensemble with a 2 fs time step. The conformational snapshots were saved for further analysis every 40 ps. For the short MD simulations of twenty models, the simulation time was 100 ns for each system (total 2 μs). For the long MD simulations of M1, M5, M10, M15, and M20, the systems were heated from 300 K to 500 K, in order to increase the reaction rate. The time of MD simulation per system was 420 ns. The heavy atoms of N-terminal domains and mismatched dsDNA were restrained by a harmonic restraint of 1 kcal·mol${}^{-1}$Å${}^{-1}$, because we mainly cared about the movement of the C-terminal domains in this study.

### 3.3. Conformational and Environmental Analysis

The root mean square deviation (RMSD), principal component analysis (PCA), distance between two atoms, angle between two C-terminal domains, and average structure analysis were done by the CPPTRAJ module of AMBERTOOLS16 [41]. Hydrogen bonds were defined with a distance cutoff of 3.5 Å between two heavy atoms and an angle cutoff of 120${}^{\circ }$ for acceptor-hydrogen-donor. The hydrogen bonds were characterized by the percentage of trajectory during which they were observed. The electrostatic surface potential of EndoMS/NucS analysis, trajectory visualization, and the corresponding figures were done using the Chimera software [37]. The calculation of velocity for all K${}^{+}$ ions during MD simulations was done by a shell script written by our group.

### 3.4. Free Energy Calculations

The C-terminal domains of EndoMS/NucS have to move about 40 Å and rotate by about 100${}^{\circ }$ from the open state (M1) to the closed state (M20). The free-energy landscape determines the conformational changes and interactions of proteins. Therefore, it is necessary to exploring the nature of relevant free-energy landscape to uncover the mechanism of protein conformational changes. In this study, the Molecular Mechanics Generalized Born Surface Area (MM-GBSA) method [48,49], which has been implemented in AMBER16, was used to calculate the free-energy landscape and the binding free energy. The free energy was estimated by the following equation:

(1) $$\Delta \Delta G_{TOT}=\Delta E_{MM}+\Delta G_{sol}-T\Delta S,$$

where ΔΔG${}_{TOT}$ is the binding free energy of the system; and ΔE${}_{MM}$, ΔG${}_{sol}$, and −TΔS are the molecular mechanics free energy, the solvation free energy, and the conformational entropy in the gas, respectively. The molecular mechanics free energy can be further divided into electrostatic interaction energy (ΔE${}_{ele}$) and van der Waals energy (ΔE${}_{vdw}$) in the gas, respectively:

(2) $$\Delta E_{MM}=\Delta E_{ele}+\Delta E_{vdw}.$$

The solvation free energy (ΔG${}_{sol}$) consists of the polar (ΔG${}_{GB}$) and nonpolar contributions (ΔG${}_{SA}$):

(3) $$\Delta G_{sol}=\Delta G_{GB}+\Delta G_{SA}.$$

The ΔG${}_{sol}$ was calculated with the GB module (IGB = 2) of the AMBER 16. The dielectric constant was set to 1.0 for the interior solute and 80.0 for the exterior solvent. The same atomic radii and charges to the MD simulations were used to calculate the binding energy. The nonpolar contribution of the solvation free energy (ΔG${}_{SA}$) was determined according to the following equation:

(4) $$\Delta G_{SA}=\gamma \times SASA+\beta ,$$

where the Solvent-Accessible Surface Area (SASA) was estimated by the MSMS algorithm with a solvent probe radius of 1.4 Å. The empirical constants γ and β were set to 0.005 kcal/(mol·Å${}^{2}$) and 0.0, respectively. The entropy term (−TΔS) was estimated by a normal mode analysis with the NMODE module in the AMBER16. The entropy calculation was only performed for the binding free energy calculations. The decomposition of binding free energy was also calculated by the MM-GBSA module.

## 4. Conclusions

In this study, extensive MD simulations have been performed, along with molecular docking, molecular modeling, and energy calculations, to explore the recognition and binding mechanism of EndoMS/NucS to mismatched dsDNA. The energy curve of EndoMS/NucS revealed that there is an energy barrier (activation angle) between the open state and the closed state. When the angle is larger than the activation angle, the C-terminal domains can freely move and prefer to converge to the open state. Otherwise, the C-terminal domains will interact with the mismatched dsDNA directly and converge to the closed state at the global energy minimum. The longer MD simulations further confirmed that the C-terminal domains can easily interact with the mismatched dsDNA and then transition to the closed state. It was also found that the change of binding state from the open state to the closed state has no effect on the interaction of the N-terminal domains with the mismatched dsDNA. The electrostatic interactions, hydrogen bonds, and π–π interactions between the N-terminal domains and the mismatched dsDNA play a critical role in recognizing and anchoring the mismatched dsDNA. The aromatic nucleus, with the two side-chains of Tyr41 and Trp77 (Tyr41${}^{\prime }$ and Trp77${}^{\prime }$), can form a binding site to recognize the mismatched bases and anchor the dsDNA, where the mismatched bases are flipped out from the DNA double helix due to the weak interaction. In addition, a new K${}^{+}$ binding site between the middle of two mismatched bases has been revealed. The binding of K${}^{+}$ can increase the stability of EndoMS/NucS-dsDNA by eliminating unfavorable interactions of the negative charges carried by the side-chains of Glu73 and Glu73${}^{\prime }$. The present study is expected to be beneficial for understanding the recognition and binding mechanisms of EndoMS/NucS in the novel DNA MMR pathway of archaeal species.

## Figures and Tables

**Table 1:** Binding free energy (kcal/mol) components for M1 and M20. ΔE${}_{ele}$, electrostatic energy in the gas phase; ΔE${}_{vdw}$, van der Waals energy; ΔG${}_{SA}$, non-polar solvation energy; ΔG${}_{GB}$, polar solvation energy; ΔG${}_{TOT}$ = ΔE${}_{ele}$ + ΔE${}_{ele}$ + ΔG${}_{GB}$ + ΔG${}_{SA}$; TΔS, entropy contribution; ΔΔG${}_{TOT}$ = ΔG${}_{TOT}$− TΔS.

Compoment	ΔE${}_{\mathrm{vdw}}$	ΔE${}_{\mathrm{ele}}$	ΔG${}_{\mathrm{SA}}$	ΔG${}_{\mathrm{GB}}$	ΔG${}_{\mathrm{TOT}}$	−TΔS	ΔΔG${}_{\mathrm{TOT}}$
**M1**	−112.32	−2512.7	−8.76	2513.51	−120.16	51.91	−68.25
**M20**	−265.49	−3777	−23.47	3858.14	−207.77	84.83	−122.94

**Table 2:** Hydrogen bonds between EndoMS/NucS and dsDNA of the N-terminal domains for M1 and M20 in the last 20 ns.

Acceptor	DonorH	Donor	Occupancy (%)	Distance (Å)
**M1 (the open state) N-terminal**				
**DG_10’@OP2**	ARG_44@HH12	ARG_44@NH1	100	2.78
**DG_8’@O6**	TRP_77@H	TRP_77@N	100	2.93
**DT_8@O4**	TRP_77’@H	TRP_77’@N	99.8	2.97
**DT_10@OP1**	TRP_77’@HE1	TRP_77’@NE1	99	2.81
**DG_8’@O6**	ASN_76@HD22	ASN_76@ND2	99	2.89
**DG_10’@OP1**	ARG_44@HH22	ARG_44@NH2	98.8	2.88
**DT_8@O4**	ASN_76’@HD22	ASN_76’@ND2	97.2	2.99
**DG_10’@OP1**	TRP_77’@HE1	TRP_77’@NE1	87.8	2.87
**DT_11’@OP1**	ARG_72’@H	ARG_72’@N	86.4	2.85
**DT_8@O2**	ARG_98’@HH22	ARG_98’@NH2	77.8	2.80
**DC_11@OP1**	GLU_73@H	GLU_73@N	74.4	3.00
**M20 (the closed state) N-terminal**				
**DG_10’@OP2**	ARG_44@HH12	ARG_44@NH1	100	2.77
**DG_10’@OP1**	ARG_44@HH22	ARG_44@NH2	100	2.88
**DG_8’@O6**	TRP_77@H	TRP_77@N	100	2.96
**DT_10@OP2**	ARG_44’@HH12	ARG_44’@NH1	99.8	2.80
**DT_10@OP1**	TRP_77’@HE1	TRP_77’@NE1	99.6	2.83
**DT_8@O4**	TRP_77’@H	TRP_77’@N	99.6	2.99
**DG_8’@O6**	ASN_76@HD22	ASN_76@ND2	99	2.91
**DT_10@OP1**	ARG_44’@HH22	ARG_44’@NH2	98.2	2.93
**DT_8@O4**	ASN_76’@HD22	ASN_76’@ND2	97.6	2.97
**DT_11’@OP1**	ARG_72’@H	ARG_72’@N	94.2	2.83
**DG_10’@OP1**	TRP_77@HE1	TRP_77@NE1	91	2.84
**DG_9@OP1**	ARG_44’@HH11	ARG_44’@NH1	78.6	2.99

## Supplementary Materials

The following are available online at https://www.mdpi.com/1422-0067/20/20/5142/s1, Figure S1: Interaction spectra between EndoMS/NucS and the mismatched dsDNA. Figure S2: Decomposition of binding free energy, on a per-residue basis, into contributions from the sum of electrostatic interactions and polar solvation energy, the van der Waals energy, and nonpolar solvation energy for the key residues of C-terminal domains of M20. Figure S3: The distance of two Mg${}^{2+}$ ions to the mismatched dsDNA: (a) Mg${}^{2+}$, and (b) Mg’${}^{2+}$. (c) The binding site of Mg${}^{2+}$. (d) The binding site of Mg’${}^{2+}$. Table S1: The binding energy contributions of the key residues of M1 and M20. Table S2: Hydrogen bonds between EndoMS/NucS and dsDNA of the C-terminal domains of M20 in the last 20 ns. Table S3: Twenty models from the open state (M1) to the closed state (M20).

## References

[1] Jackson S.P., Bartek J. (2009). The DNA-damage response in human biology and disease. Nature.

[2] Helena J.M., Joubert A.M., Grobbelaar S., Nolte E.M., Nel M., Pepper M.S., Coetzee M., Mercier A.E. (2018). Deoxyribonucleic Acid Damage and Repair: Capitalizing on Our Understanding of the Mechanisms of Maintaining Genomic Integrity for Therapeutic Purposes. Int. J. Mol. Sci..

[3] Barnes D.E., Lindahl T. (2004). Repair and genetic consequences of endogenous DNA base damage in mammalian cells. Annu. Rev. Genet..

[4] Iyer R.R., Pluciennik A., Burdett V., Modrich P.L. (2006). DNA mismatch repair: Functions and mechanisms. Chem. Rev..

[5] Skucha A., Ebner J., Grebien F. (2019). Roles of SETD2 in LeukemiaTranscription, DNA-Damage, and Beyond. Int. J. Mol. Sci..

[6] Gomes L.R., Menck C.F.M., Leandro G.S. (2017). Autophagy Roles in the Modulation of DNA Repair Pathways. Int. J. Mol. Sci..

[7] Kim J.H. (2019). Chromatin Remodeling and Epigenetic Regulation in Plant DNA Damage Repair. Int. J. Mol. Sci..

[8] Kunkel T.A., Erie D.A. (2005). DNA mismatch repair. Annu. Rev. Biochem..

[9] Eisen J.A., Hanawalt P.C. (1999). A phylogenomic study of DNA repair genes, proteins, and processes. Mutat. Res.-DNA Repair.

[10] Lenhart J.S., Schroeder J.W., Walsh B.W., Simmons L.A. (2012). DNA Repair and Genome Maintenance in Bacillus subtilis. Microbiol. Mol. Biol. Rev..

[11] Su S.S., Modrich P. (1986). Escherichia coli mutS-encoded protein binds to mismatched DNA base pairs. Proc. Natl. Acad. Sci. USA.

[12] Lee H., Popodi E., Tang H.X., Foster P.L. (2012). Rate and molecular spectrum of spontaneous mutations in the bacterium Escherichia coli as determined by whole-genome sequencing. Proc. Natl. Acad. Sci. USA.

[13] Putnam C.D. (2016). Evolution of the methyl directed mismatch repair system in Escherichia coli. DNA Repair.

[14] Castaneda-Garcia A., Prieto A.I., Rodriguez-Beltran J., Alonso N., Cantillon D., Costas C., Perez-Lago L., Zegeye E.D., Herranz M., Plocinski P. (2017). A non-canonical mismatch repair pathway in prokaryotes. Nat. Commun..

[15] Ishino S., Nishi Y., Oda S., Uemori T., Sagara T., Takatsu N., Yamagami T., Shirai T., Ishino Y. (2016). Identification of a mismatch-specific endonuclease in hyperthermophilic Archaea. Nucleic Acids Res..

[16] Banasik M., Sachadyn P. (2014). Conserved motifs of MutL proteins. Mutat. Res.-Fundam. Mol. Mech. Mutagen..

[17] Ford C.B., Lin P.L., Chase M.R., Shah R.R., Iartchouk O., Galagan J., Mohaideen N., Ioerger T.R., Sacchettini J.C., Lipsitch M. (2011). Use of whole genome sequencing to estimate the mutation rate of Mycobacterium tuberculosis during latent infection. Nat. Genet..

[18] Lin Z., Nei M., Ma H. (2007). The origins and early evolution of DNA mismatch repair genesmultiple horizontal gene transfers and co-evolution. Nucleic Acids Res..

[19] Grogan D.W., Carver G.T., Drake J.W. (2001). Genetic fidelity under harsh conditions: Analysis of spontaneous mutation in the thermoacidophilic archaeon Sulfolobus acidocaldarius. Proc. Natl. Acad. Sci. USA.

[20] Grogan D.W. (2004). Stability and repair of DNA in hyperthermophilic archaea. Curr. Issues Mol. Biol..

[21] Busch C.R., DiRuggiero J. (2010). MutS and MutL Are Dispensable for Maintenance of the Genomic Mutation Rate in the Halophilic Archaeon Halobacterium salinarum NRC-1. PLoS ONE.

[22] Ishino S., Skouloubris S., Kudo H., I’Hermitte-Stead C., Es-Sadik A., Lambry J.C., Ishino Y., Myllykallio H. (2018). Activation of the mismatch-specific endonuclease EndoMS/NucS by the replication clamp is required for high fidelity DNA replication. Nucleic Acids Res..

[23] Takemoto N., Numata I., Su’etsugu M., Miyoshi-Akiyama T. (2018). Bacterial EndoMS/NucS acts as a clamp-mediated mismatch endonuclease to prevent asymmetric accumulation of replication errors. Nucleic Acids Res..

[24] Nakae S., Hijikata A., Tsuji T., Yonezawa K., Kouyama K., Mayanagi K., Ishino S., Ishino Y., Shirai T. (2016). Structure of the EndoMS-DNA Complex as Mismatch Restriction Endonuclease. Structure.

[25] Ariyoshi M., Morikawa K. (2016). A Dual Base Flipping Mechanism for Archaeal Mismatch Repair. Structure.

[26] Creze C., Lestini R., Kuhn J., Ligabue A., Becker H.F., Czjzek M., Flament D., Myllykallio H. (2011). Structure and function of a novel endonuclease acting on branched DNA substrates. Biochem. Soc. Trans..

[27] Fishel R., Ewel A., Lescoe M.K. (1994). Purified human MSH2 protein binds to DNA containing. Cancer Res..

[28] Su S.S., Lahue R.S., Au M.K., Modrich P. (1988). Mispair specificity of methyl-directed DNA mismatch correction in vitro. Biol. Chem..

[29] Natrajan G., Lamers M.H., Enzlin J.H., Winterwerp H.K., Perrakis A., Sixma T.K. (2003). Structure of Escherichia coli DNA mismatch repair enzyme MutS in complex with different mismatches: a common recognition mode for diverse substrates. Nucleic Acids Res..

[30] Kok D.B., Groothuizen F.S., Fish A., Dharadhar S., Winterwerp H.K., Sixma T.K. (2019). Sharp kinking of a coiled-coil in MutS allows DNA binding and release. Nucleic Acids Res..

[31] Sali A., Blundell T.L. (1993). Comparative protein modelling by satisfaction of spatial restraints. J. Mol. Biol..

[32] Huang S.-Y. (2015). Exploring the potential of global protein-protein docking: An overview and critical assessment of current programs for automatic ab initio docking. Drug Discov. Today.

[33] Huang S.-Y., Zou X. (2014). A knowledge-based scoring function for protein-RNA interactions derived from a statistical mechanics-based iterative method. Nucleic Acids Res..

[34] Yan Y., Wen Z., Wang X., Huang S.-Y. (2017). Addressing recent docking challenges: A hybrid strategy to integrate template-based and free protein-protein docking. Proteins.

[35] Yan Y., Zhang D., Zhou P., Li B., Huang S.-Y. (2017). HDOCK: A web server for protein-protein and protein-DNA/RNA docking based on a hybrid strategy. Nucleic Acids Res..

[36] He J., Tao H., Huang S.-Y. (2019). Protein-ensemble-RNA docking by efficient consideration of protein flexibility through homology models. Bioinformatics.

[37] Pettersen E.F., Goddard T.D., Huang C.C., Couch G.S., Greenblatt D.M., Meng E.C., Ferrin T.E. (2004). UCSF chimera—A visualization system for exploratory research and analysis. J. Comput. Chem..

[38] Anandakrishnan R., Aguilar B., Onufriev A.V. (2012). H++3.0: automating pK prediction and the preparation of biomolecular structures for atomistic molecular modeling and simulations. Nucleic Acids Res..

[39] Gordon J.C., Myers J.B., Folta T., Shoja V., Heath L.S., Onufriev A. (2005). H++: A server for estimating pK(a)s and adding missing hydrogens to macromolecules. Nucleic Acids Res..

[40] Myers J., Grothaus G., Narayanan S., Onufriev A. (2006). A simple clustering algorithm can be accurate enough for use in calculations of pKs in macromolecules. Proteins.

[41] Case D.A., Betz R.M., Cerutti D.S., Cheatham T.E., Darden T.A., Duke R.E., Giese T.J., Gohlke H., Goetz A.W., Nadine H. (2016). AMBER 2016.

[42] Jorgensen W.L., Chandrasekhar J., Madura J.D., Impey R.W., Klein M.L. (1983). Comparison of simple potential functions for simulating liquid water. J. Chem. Phys..

[43] Tom Darden D.Y., Lee P. (1993). Particle mesh Ewald: An N·log(N) method for Ewald sums in large systems. J. Chem. Phys..

[44] Ryckaert J.-P., Ciccotti G., Berendsen H.J.C. (1977). Numerical integration of the cartesian equations of motion of a system with constraints: Molecular dynamics of n-alkanes. J. Comput. Phys..

[45] Zhang Y.J., Ding J.N., Zhong H., Han J.G. (2017). Exploration micromechanism of VP35 IID interaction and recognition dsRNA: A molecular dynamics simulation. Proteins.

[46] Zhang Y.J., Ding J.N., Feng T.T., Han J.G. (2015). Exploring interaction mechanisms of the inhibitor binding to the VP35 IID region of Ebola virus by all atom molecular dynamics simulation method. Proteins.

[47] Zhang Y.J., Tao H.Y., Huang S.Y. (2019). Dynamics and Mechanisms in the Recruitment and Transference of Histone Chaperone CIA/ASF1. Int. J. Mol. Sci..

[48] Kollman P.A., Massova I., Reyes C., Kuhn B., Huo S.H., Chong L., Lee M., Lee T., Duan Y., Wang W. (2000). Calculating structures and free energies of complex molecules: Combining molecular mechanics and continuum models. Accounts Chem. Res..

[49] Homeyer N., Gohlke H. (2012). Free Energy Calculations by the Molecular Mechanics Poisson-Boltzmann Surface Area Method. Mol. Inf..

