# Supplementary material for: Exploring the Binding Mechanism and Dynamics of EndoMS/NucS to Mismatched dsDNA
Source: Int J Mol Sci. 2019 Oct 17;20(20):5142. doi: 10.3390/ijms20205142 (PMC6829318; doi:10.3390/ijms20205142)
Supplement: Supplementary file 1 [file ijms-20-05142-s001.pdf]

Figure S1 The interaction spectra between EndoMS/NucS and the mismatched dsDNA .

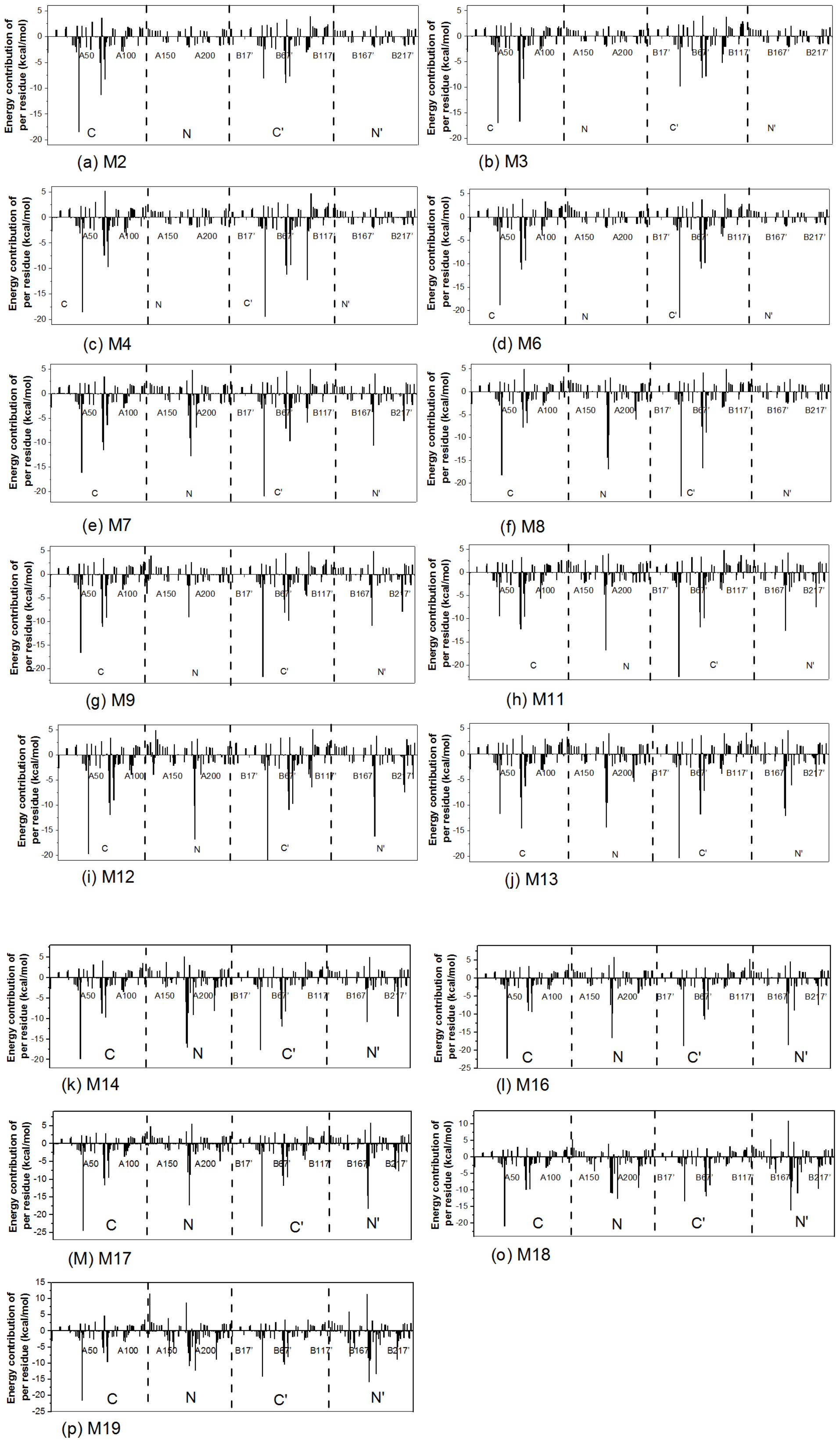

Figure S2 Decomposition of binding free energy on per-residue basis into contributions from the sum of electrostatic interactions and polar solvation energy ($\Delta G_{ele} = \Delta E_{ele} + \Delta G_{GB}$), the van der Waals energy (ΔE_{vdw}), and nonpolar solvation energy (ΔG_{SA}) for the key residues of C-terminal domains of M20.

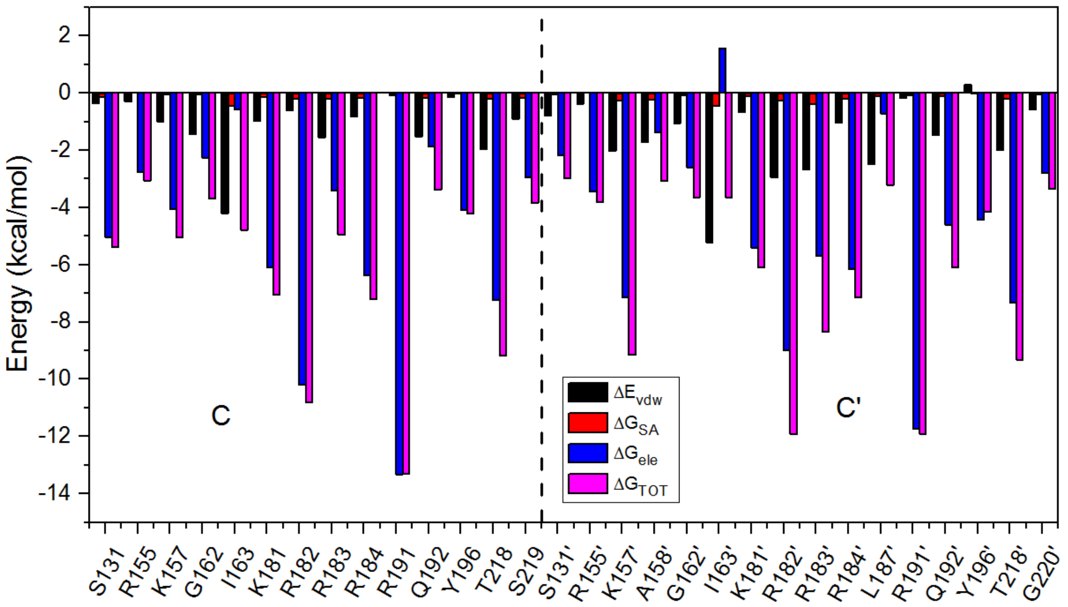

Figure S3 The distance of two Mg^{2+} ions to the mismatched dsDNA: (a) Mg, (b) Mg'. (c) The binding site of Mg^{2+}. (d) The binding site of Mg'^{2+}.

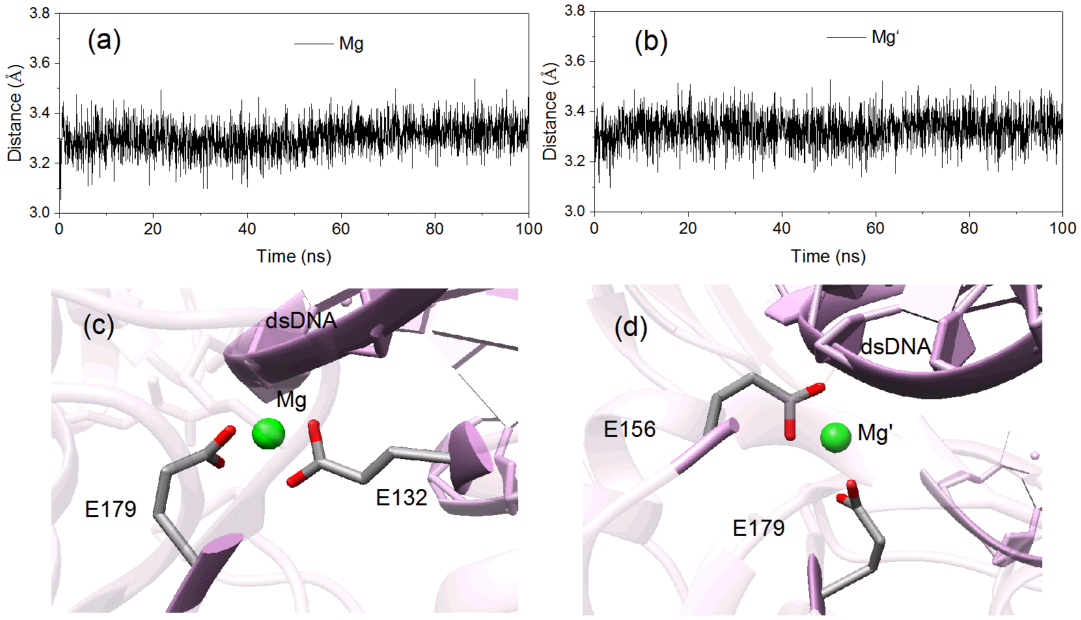

Table S1 The binding energy contributions of key residues of M1 and M20. Vdw van der Waals energy; ele, electrostatic energy; GB, polar solvation energy; SA, non-polar solvation energy; S, side-chain atoms; B, back-bone atoms; T, total energy. All values are given in kcal/mol.

Rsidues	T_{vdw}	S_{ele}	B_{ele}	T_{ele}	T_{GB}	T_{SA}	T_{GBTOT}
**M1 (The open state)**							
**Y41**	-3.5	-5.01	4	-1	1.61	-0.11	-3
**R44**	-3.75	-261.16	-58.58	-319.74	304.64	-0.43	-19.27
**K71**	-1.68	-231.52	-64.42	-295.94	289.83	-0.28	-8.06
**R72**	-3.32	-233.71	-72.03	-305.74	298.61	-0.2	-10.65
**N76**	-0.59	-11.73	-2.91	-14.64	10.73	-0.04	-4.54
**W77**	-4.7	-8.77	-4.46	-13.23	8.87	-0.34	-9.39
**K100**	-0.61	-163.33	-42.52	-205.85	203.41	-0.19	-3.24
**R44'**	-3.24	-230.12	-58.31	-288.43	284.32	-0.34	-7.69
**K71'**	-2	-231.63	-65.91	-297.54	290.29	-0.33	-9.58
**R72'**	-2.81	-233.36	-71.68	-305.04	296.54	-0.17	-11.49
**N76'**	-0.67	-10.92	-3.08	-14	11.29	-0.04	-3.42
**W77'**	-5.19	-11.34	-0.03	-11.37	7.26	-0.41	-9.72
**R98'**	-0.21	-214.69	-49.72	-264.41	259.42	-0.07	-5.27
**K100'**	-0.36	-175.4	-40.15	-215.55	212.89	-0.15	-3.17
**M20 (The closed state)**							
**Y41**	-3.63	-4.94	4.2	-0.73	1.42	-0.1	-3.04
**R44**	-3.62	-259.98	-59.5	-319.47	302.6	-0.29	-20.78
**K71**	-1.84	-231.56	-66.66	-298.22	291.29	-0.37	-9.14
**R72**	-3.2	-230.79	-72.8	-303.59	296.04	-0.2	-10.95
**N76**	-0.54	-12.13	-2.98	-15.11	11.05	-0.03	-4.64
**W77**	-5	-9.02	-4.53	-13.55	9.22	-0.35	-9.68
**R98**	-0.84	-213.12	-48.65	-261.77	259.59	-0.03	-3.04
**S131**	-0.35	-14.28	-4.38	-18.66	13.75	-0.13	-5.39
**R155**	-0.3	-172.05	-51.26	-223.31	220.55	0	-3.05
**K157**	-0.99	-155.02	-52.21	-207.24	203.24	-0.06	-5.04
**G162**	-1.43	7.03	-19.41	-12.38	10.18	-0.06	-3.69
**I163**	-4.2	-8.1	10.1	2	-2.14	-0.43	-4.78
**K181**	-0.96	-251.37	-62.13	-313.5	307.54	-0.13	-7.05
**R182**	-0.6	-238.98	-62.65	-301.63	291.65	-0.21	-10.79
**R183**	-1.54	-250.35	-61.61	-311.96	308.76	-0.21	-4.95
**R184**	-0.83	-198.53	-52.41	-250.94	244.75	-0.18	-7.2
**R191**	0.03	-239.77	-60.18	-299.95	286.69	-0.07	-13.31
**Q192**	-1.53	-8.29	3.01	-5.28	3.58	-0.16	-3.38
**Y196**	-0.13	-13.51	1.8	-11.71	7.63	-0.02	-4.22
**T218**	-1.94	-14.17	-7.27	-21.43	14.38	-0.19	-9.18
**S219**	-0.9	-8.47	-2.68	-11.15	8.37	-0.16	-3.84

**R44'**	-3.46	-264.19	-58.22	-322.41	304.79	-0.36	-21.44
**K71'**	-2.02	-230.53	-65.91	-296.43	289.16	-0.33	-9.62
**R72'**	-2.92	-231.58	-70.99	-302.57	294.44	-0.18	-11.23
**N76'**	-0.6	-10.51	-2.48	-12.99	9.8	-0.03	-3.82
**W77'**	-5.3	-11.4	-0.37	-11.77	7.93	-0.41	-9.56
**R98'**	-0.39	-210.51	-47.07	-257.58	254.91	-0.01	-3.07
**K100'**	-0.26	-181	-45.37	-226.37	223.46	-0.15	-3.32
**S131'**	-0.8	-9.41	-3.32	-12.73	10.61	-0.05	-2.97
**R155'**	-0.37	-191.63	-49.28	-240.91	237.46	0	-3.82
**K157'**	-2.02	-198.63	-57.82	-256.46	249.6	-0.27	-9.15
**A158'**	-1.72	-8.24	0.83	-7.42	6.28	-0.22	-3.08
**G162'**	-1.05	7.21	-17.44	-10.23	7.72	-0.08	-3.64
**I163'**	-5.22	-8.53	12.47	3.94	-1.92	-0.45	-3.65
**K181'**	-0.66	-246.21	-64.23	-310.43	305.13	-0.12	-6.09
**R182'**	-2.94	-233	-67.45	-300.45	291.73	-0.27	-11.92
**R183'**	-2.67	-264.62	-61.04	-325.66	320.36	-0.38	-8.35
**R184'**	-1.02	-198.25	-52.44	-250.69	244.75	-0.2	-7.15
**L187'**	-2.49	-7.19	-0.01	-7.2	6.61	-0.12	-3.21
**R191'**	-0.17	-235.46	-60.62	-296.08	284.42	-0.07	-11.9
**Q192'**	-1.46	-12.69	3.79	-8.9	4.41	-0.12	-6.08
**Y196'**	0.29	-14.46	2.16	-12.29	7.87	-0.01	-4.15
**T218'**	-2	-12.33	-6.75	-19.09	11.97	-0.2	-9.31
**G220'**	-0.56	6.61	-19.57	-12.97	10.21	-0.04	-3.35

Table S2 Hydrogen bonds between EndoMS/NucS and dsDNA of the C-terminal domains for M20 in the last 20 ns.

**Acceptor**	**DonorH**	**Donor**	**Occupancy (%)**	**Distance (Å)**
**M20 (The closed state) C-terminal**				
DA_6'@OP2	GLN_192'@HE21	GLN_192'@NE2	0.994	2.9214
DT_9'@OP1	ARG_183'@HH11	ARG_183'@NH1	0.984	2.9238
DT_9'@OP2	ARG_182'@HH21	ARG_182'@NH2	0.982	2.8112
DA_3'@OP2	ARG_184@HH11	ARG_184@NH1	0.976	2.8203
DC_3@OP2	ARG_184'@HH11	ARG_184'@NH1	0.972	2.8336
DA_7@OP1	GLU_132@H	GLU_132@N	0.956	2.9358
DA_8@OP1	SER_219@H	SER_219@N	0.94	2.8945
DC_7'@OP1	GLU_132'@H	GLU_132'@N	0.926	3.1373
DA_5@OP1	ILE_163@H	ILE_163@N	0.92	3.0509
DA_5@OP1	ARG_191'@HH12	ARG_191'@NH1	0.912	2.9234
DG_5'@OP2	ARG_191@HH12	ARG_191@NH1	0.886	2.8762
DG_9@OP2	ARG_182@HH12	ARG_182@NH1	0.882	2.7725
DA_5@OP2	ARG_191'@HH22	ARG_191'@NH2	0.882	2.8636
DC_3@OP1	SER_219'@H	SER_219'@N	0.864	2.9519
DG_9@OP2	ARG_182@HH22	ARG_182@NH2	0.808	2.8178
DG_5'@OP1	ILE_163'@H	ILE_163'@N	0.786	3.0864
DC3_15@OP1	ALA_158'@H	ALA_158'@N	0.774	3.0329
DG_5@OP1	ARG_191@HH22	ARG_191@NH2	0.76	2.925

Table S3 Twenty models from the open state (M1) to the closed state (M20).

Model	Angle(°)	Model	Angle(°)	Model	Angle(°)
**M1**	117	**M8**	71	**M15**	36
**M2**	109	**M9**	66	**M16**	28
**M3**	101	**M10**	63	**M17**	25
**M4**	95	**M11**	55	**M18**	17
**M5**	89	**M12**	50	**M19**	12
**M6**	84	**M13**	47	**M20**	10
**M7**	76	**M14**	42		
